# Attentional bias in math anxiety

**DOI:** 10.3389/fpsyg.2015.01539

**Published:** 2015-10-16

**Authors:** Orly Rubinsten, Hili Eidlin, Hadas Wohl, Orly Akibli

**Affiliations:** Edmond J. Safra Brain Research Center for the Study of Learning Disabilities, Department of Learning Disabilities, University of HaifaHaifa, Israel

**Keywords:** math anxiety, dot probe, attentional bias

## Abstract

Cognitive theory from the field of general anxiety suggests that the tendency to display attentional bias toward negative information results in anxiety. Accordingly, the current study aims to investigate whether attentional bias is involved in math anxiety (MA) as well (i.e., a persistent negative reaction to math). Twenty seven participants (14 with high levels of MA and 13 with low levels of MA) were presented with a novel computerized numerical version of the well established dot probe task. One of six types of prime stimuli, either math related or typically neutral, was presented on one side of a computer screen. The prime was preceded by a probe (either one or two asterisks) that appeared in either the prime or the opposite location. Participants had to discriminate probe identity (one or two asterisks). Math anxious individuals reacted faster when the probe was at the location of the numerical related stimuli. This suggests the existence of attentional bias in MA. That is, for math anxious individuals, the cognitive system selectively favored the processing of emotionally negative information (i.e., math related words). These findings suggest that attentional bias is linked to unduly intense MA symptoms.

## Introduction

Mathematical skills are essential for productive functioning in our progressively more complex, technological society. In addition, numerical development has been a focus of the continuing theoretical debate concerning the origins of cognition and how it develops throughout one’s lifetime. Numerical difficulties result in reduced educational and employment achievements, and in increased physical and mental health costs (Woloshin et al., 2001; Parsons and Bynner, 2005; Duncan et al., 2007; Reyna et al., 2009). Some argue that in western society, poor numeracy is a greater handicap than poor literacy (e.g., Rivera-Batiz, 1992; Estrada et al., 2004). Hence, mathematical skills may have an impact on social mobility and poverty levels.

However, some people find it difficult to learn arithmetic or mathematics since they suffer from math anxiety (henceforth math anxiety, or MA), which is a persistent negative reaction to math, ranging from mild discomfort to extreme avoidance (Hembree, 1990; Ma and Xu, 2004a,b; Ashcraft and Ridley, 2005). Given the implications of MA, a systematic identification of the vulnerability factors that contribute to the development and maintenance of MA is crucial. But what are these possible vulnerability factors? According to information processing theories, fear and anxiety may be caused by different cognitive processes, such as attention. Compared to non-anxious individuals, anxious individuals are more likely to show an inclination to attend to threatening stimuli over non-threatening stimuli in their environment (attentional bias) (for review see Van Bockstaele et al., 2014). Attentional bias to threatening stimuli was found for general, but not MA. The current study aims to fill this gap.

Math anxiety consists of feelings of tension (Richardson and Suinn, 1972) and low self confidence in one’s ability to learn mathematics (Jain, 2009). In addition, MA can affect general cognitive abilities such as decline in working memory (Ashcraft and Kirk, 2001). Cognitive causes may also involve core numerical characteristics such as counting abilities (Maloney et al., 2010), the precision of the mental number line (Maloney et al., 2011), and poor numeracy (i.e., the ability to estimate large quantities of items – Rubinsten and Tannock, 2010). MA was also found to have a possible genetic (Wang et al., 2014) and a specific neural basis (Young et al., 2012), even when only anticipating math problems (Lyons and Beilock, 2011); this was found in the bilateral inferior frontal junction, a brain region known to be involved in cognitive control and reappraisal of negative emotional responses. The more highly math anxious individuals activated this frontoparietal network before they even engaged in mathematics; the better they performed on a math task.

In terms of epidemiology, recent findings show that even children as young as the first grade suffer from MA (Ramirez et al., 2013). In addition, although there are exceptions, most studies of MA report higher levels of MA for females than for males (e.g., Betz, 1978; Hembree, 1990; Ashcraft and Faust, 1994; Hopko, 2003; Ma and Cartwright, 2003; Haynes et al., 2004; Baloglu and Kocak, 2006; McGraw et al., 2006; Jain, 2009; Else-Quest et al., 2010). However, other studies failed to find such a gender difference (e.g., Cooper and Robinson, 1991). These gender differences appear despite the fact that no difference is typically found between genders in math knowledge and skills (for a meta-analysis see Else-Quest et al., 2010).

Even mild levels of MA have been associated with academic decisions (Brown et al., 2010). This may suggest that MA may be a strong antecedent for the low visibility of women in the science and engineering workforce. For example, despite gender similarities in math achievements (Hedges and Nowell, 1995; Hyde et al., 2008; Else-Quest et al., 2010) or even better math grades for females compared to males (Kenney-Benson et al., 2006), in the US women constitute only 28% of the science and engineering workforce (correct for the year of 2010 – National Science Foundation, 2013). Women are also severely underrepresented in math-intensive fields (Ceci and Williams, 2011). Hence, as our society becomes progressively more dependent on math, failure to acquire numerical skills may increasingly act as a filter, preventing occupational success for men but mainly for women (e.g., Halpern et al., 2007). This makes it a very good reason to study MA.

The current study aims to investigate the cognitive source of MA. It is still quite rare to see cognitive neuroscience research take into account issues of MA, and only scant attention has been devoted to the antecedents of MA. By suggesting the role played by anxiety in numerical situations, scientists and clinicians will be better able to provide cognitive models of both MA vulnerability and math dysfunction.

As mentioned above, the antecedents and epidemiology of MA are still being studied and results are inconclusive. One variable that might be related to different findings regarding MA is the common use of explicit tools such as the MA rating scale (e.g., Richardson and Suinn, 1972), the MA questionnaire (Wigfield and Meece, 1988) (for a German version see Krinzinger et al., 2007), the abbreviated math anxiety scale (AMAS: Hopko et al., 2003), or the revised Math Anxiety Rating Scale (MARS-R: Alexander and Martray, 1989; Hopko, 2003) to diagnose MA. Such explicit tools typically assess accessible self representations.

However, women, for example, have been found to score higher than men on self-report measures of trait anxiety (e.g., Feingold, 1994; Costa et al., 2001; Egloff and Schmukle, 2004), possibly resulting from gender differences in anxiety that are not due to anxiety *per se*. That is, gender differences in explicit self-report questionnaires could be the result of greater willingness of women to disclose personal attitudes (Ashcraft, 2002). Indeed, Flessati and Jamieson (1991) argued that gender differences in MA could be explained by the fact that females are more self- critical of their performance.

Implicit measures, on the other hand, typically assess inaccessible cognitive structures or representations that are processed automatically. It has been shown that affective traits can be activated automatically and influence emotional, cognitive, and behavioral processes (e.g., Giner-Sorolla et al., 1999) even in the case of MA (Rubinsten et al., 2012). That is, affective processing begins immediately and even involuntarily upon seeing a salient affective word or picture (for review see Rubinsten, 2015).

Thus, one of our primary objectives is to investigate cognitive characteristics of MA, and specifically attentional bias, by using a novel attention bias task as an indirect measure.

Math anxiety has been found to be positively, albeit moderately, correlated with general, state, and trait anxiety (Ashcraft and Moore, 2009). General anxiety is traditionally classified into two distinct components, “trait” and “state.” While trait anxiety refers to relatively stable individual differences in anxiety proneness, state anxiety is a transitory emotional condition (Spielberger and Spielberger, 1966). Mathematics anxiety is conceptualized as a situation (i.e., trait) specific anxiety that manifests itself in mathematics-related environments (e.g., Baloglu, 1999). These similarities between general and MA, may suggest that the cognitive traits that are associated with general anxiety, such as the tendency to ruminate over negative thoughts and stressful situations (Donaldson et al., 2007) or the tendency to display attentional bias toward negative information (Bar-Haim et al., 2007), are involved not only in general anxiety but also in MA. Interestingly, and to the best of our knowledge, contemporary scientific approaches have not availed themselves of this insight, which suggests a link between the cognitive symptoms of general and MA. Accordingly, here we wish to focus on attentional bias in MA via an implicit and novel cognitive tool.

Rumination is defined as repetitive thinking about negative personal concerns and/or about the implications and causes of a negative mood (Nolen-Hoeksema et al., 2008). Indeed, the tendency to ruminate has been associated with self-reported symptoms of generalized anxiety (Fresco et al., 2002; Harrington and Blankenship, 2002), post-traumatic stress (Nolen-Hoeksema and Morrow, 1991; Clohessy and Ehlers, 1999; Mayou et al., 2002), and social anxiety (Mellings and Alden, 2000).

Rumination affects the ability to remain attentive to the task at hand due to obsessive thoughts over negative feelings (Donaldson et al., 2007). Reese et al. (2010) have suggested that attentional bias to negative information is linked to the repetitive negative thinking characteristic of anxious rumination and worry. Indeed, rumination and attentional bias have been linked to stress and to each other (e.g., Bradley et al., 1997; Beevers and Carver, 2003; Mogg and Bradley, 2005). Morrison and O’Connor (2008) even suggested a causal relationship in which rumination affects attentional bias. Hence, clinically anxious patients have been shown to display attentional bias toward negative information (Bar-Haim et al., 2007). It has been suggested that biased patterns of information processing (such as rumination and attentional bias) operate within the cognitive system at a very early stage and hence, are unreachable to awareness and play a central causal role in susceptibility to experiencing overly intense general anxiety symptoms (Mathews and MacLeod, 2005). Another approach concerning the link between anxiety and attention is described by the attentional control theory suggested by Eysenck et al. (2007). According to the attentional control theory, the anxiety state is capable of increasing the allocation of attention to threat related stimuli. That is, anxiety typically reduces attentional focus on a current task unless it involves threatening stimuli; or in other words anxiety impairs attentional control. Therefore, we aim to examine attentional bias in MA and to suggest that it is attentional bias that leads to unduly intense MA symptoms and to damage to information processing (i.e., solving math problems). This suggestion of ours, is based on cognitive theory from the field of general anxiety (Beck et al., 1979), which posits that certain cognitive vulnerabilities (such as attentional bias), when ‘activated’ by stressful or negative life events, result in psychological distress.

Attentional bias has been assessed in various ways. One technique is the visual probe task, in which stimuli that differ in their emotional tone are briefly exposed on a computer screen before a visual probe appears in the locus where one or another emotional stimuli were exposed (Koster et al., 2006; Colin et al., 2007). Participants must quickly discriminate probe identity. Typically, responses are found to be faster when probes appear in the locus of negative stimuli. Hence, attentional bias in the dot probe task could arise from fast responding in congruent trials (attentional engagement to threat), slow responding in incongruent trials (slow attentional disengagement away from threat), or a combination of both (e.g., Koster et al., 2004). Such a pattern of results provides an index of selective attention to negative or threatening information. This dot probe task has showed attentional bias to negative stimuli in both clinical and non-clinical expressions of anxiety (Cisler and Koster, 2010).

The purposes of the current study are to strengthen MA assessment (i.e., by using an implicit instead of an explicit tool) and to focus on attention bias in MA. For that, we developed a novel computerized numerical version of the well established dot probe task (MacLeod et al., 1986), which has been proven to be a highly reliable tool in the assessment and even treatment of general anxiety (e.g., Baert et al., 2010). We hypothesized that math anxious individuals would react faster when the probe is at the location of the threat/numerical related prime (e.g., based on Bar-Haim, 2010). That is, as in the typical dot probe task, faster reaction times (RTs) when probes appear in the locus of numerical primes, will point to selective attention to negative information (i.e., attentional bias in MA).

## Materials and Methods

### Participants

Twenty-eight adults participated in the study (nine males, mean age = 26.44 years, *SD* = 4.61). One female participant was excluded due to missing data. All participants were recruited through advertisements distributed on a university campus. All participants gave their written consent to participate in the experiment and were paid about 10USD for their participation. The recruitment, payment, task, and overall procedure were authorized by the research ethics committee of the university.

#### Classification and Assessments Criteria

Participants were sorted into groups of MA as follows: high math anxiety (HMA) or low math anxiety (LMA), based on their score on the MARS-R questionnaire (Plake and Parker, 1982). The cut-off threshold for inclusion was a score below (for the LMA group) or above (for the HMA group) 72 points, which was the group median score. An independent *t*-test yielded significant differences between HMAs (14 participants of whom 4 were males, *M* = 83.4, *SD* = 10.83) and LMAs (13 participants of whom five were males, *M* = 57.9, *SD* = 11.8) on the MARS-R scores [*T*_(25)_ = 5.8, *p* < 0.001]. It is interesting to note that no gender difference was found in the MARS-R scores [*T*_(24)_ = -1.1, n.s.].

#### The Experimental Tasks and Stimuli

##### The novel numerical dot probe task

###### Stimuli

A novel dot probe task was created for the experiment, based on the method of the well established dot probe task initially developed by MacLeod et al. (1986). A prime stimuli, either math related (a math equation such as 26 + 65 or a math word such as “quantity”) or typically neutral (a word with neutral valence such as “table”), are presented on one side of a computer screen, and are then preceded by a probe (either one or two asterisks “^∗^”) that appears in either the prime location (congruent) or the opposite location (incongruent). Participants must quickly discriminate probe identity (one or two asterisks) and then preform a task regarding the prime stimuli.

One of six types of primes appeared on either the left or right side of the computer screen. There were four different equation levels. Accordingly, the prime could be either a single digit arithmetic equation (e.g., 8–4), a double digit (e.g., 52 + 16), a triple digit (e.g., 536/268), or a power equation (e.g., 9^2^ × 3^5^), math word (e.g., number), or neutral word (e.g., pencil).

Each equation level (i.e., single, double, triple digit, or power) contained four pairs of numbers (e.g., 8 and 4). Each pair of numbers produced four trials: each type of these trials involved one of the four basic operations: addition, subtraction, multiplication, or division (e.g., the pair 8 and 4 produced the equations 8 + 4, 8 - 4, 8^∗^4, and 8/4). There were three major rules for pair matching: (1) Each pair of numbers was chosen based on numerical length (either single, double, or triple digits). (2) One number in each pair was a multiplication of the other. (3) Digit frequency (1–9) was controlled across all numerical combinations (for a detailed list of the numbers, see Appendix 1).

The word stimuli consisted of 16 math related words and 16 neutral words. All words were chosen based on their frequency and emotional load. Frequency levels and emotional load were tested by a short questionnaire distributed online (by Google form document) to 58 university students. For each item participants were asked how familiars the word on a 9 point Likert scale (1- not familiar, 9- very familiar) and how frightening is the word on a 9 point Likert scale (1- not frightening at all, 9- very frightening). The words were also matched by their length, i.e., number of letters (for detailed information see Appendix 1).

The prime appeared on a black background and was positioned on one side of the computer screen at a 3.81° (short stimuli) – 16.84° (long stimuli) visual angle (VA; VA was calculated using the following formula: θ = 2tan^-1^(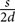) where *d* is the distance between the participant’s eye and the screen and *s* is the size of the object on the screen).

**FIGURE 1:**
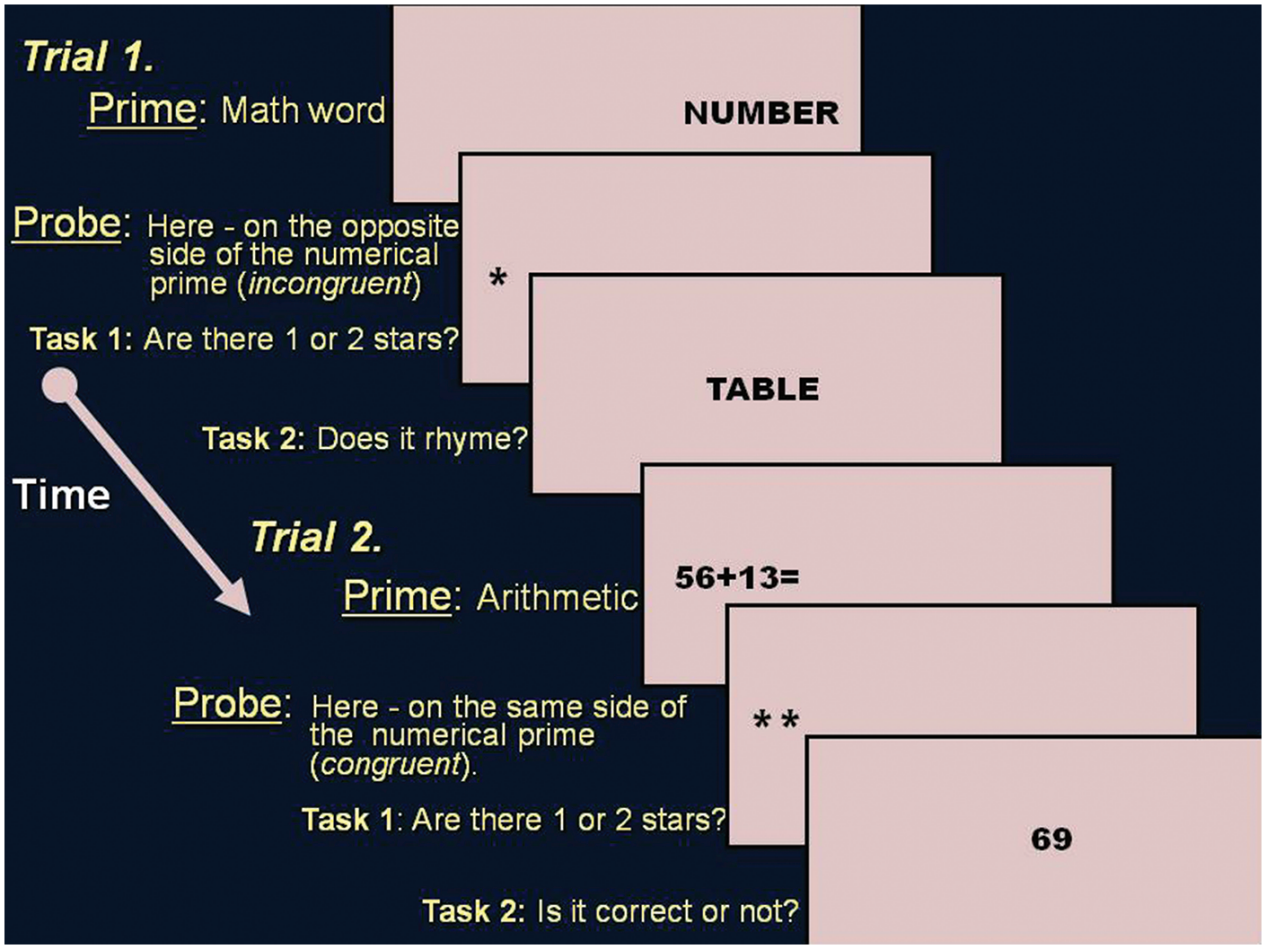
**Examples of stimuli in the numerical dot probe task**.

The prime presentation was followed by a probe identification task. The probe was either one (i.e., ^∗^) or two asterisks (i.e., ^∗∗^). The probe could appear on the same side previously occupied by the prime (i.e., congruent trial) or on the opposite side (i.e., incongruent trial). In order to avoid visual bias, the probe’s exact location was chosen randomly, so it could appear at seven different locations on each side of the screen, matching all possible locations previously occupied by the prime (either by the numbers of the math equation or the letters of the words). Participants were asked to determine if there were one or two asterisks (first task – see **Figure 1**). Following the probe identification task, and after the participant responded to the probe, the probe disappeared and either a number (after math equation prime) or a word (after word prime) appeared in the center of the computer screen. Participants were asked (second task) to determine whether the number was the correct answer to the previously presented equation (i.e., prime) or not. In cases of word prime trials, participants had to determine, in this second task, whether the word that appeared in the center of the screen rhymed with the previous word or not. This second task was presented in order to make sure that participants indeed processed the prime and to create meaningful math stimuli.

###### Procedure

Each trial in our numerical dot probe task began with a white colored square shaped fixation point, presented for 750 ms and followed by a blank screen presented for 100 ms. Then, a prime appeared on either the left or the right side of the screen and remained for 1000 ms. Next, there was an inter stimulus interval (ISI) of 100–150 ms (the exact ISI changed in between stimuli to avoid participant prediction of the stimuli’s appearance for similar rationale and ISI see e.g., Posner and Boies, 1971). Afterward, a small probe (one or two asterisks) appeared either on the side previously occupied by the prime (congruent trial) or on the opposite side of the screen (incongruent trial). Participants were instructed to determine whether one or two asterisks appeared on the computer screen by pressing one of two optional keys on the keyboard (the numbers 1, 2). Half of the participants were asked to use their right hand to respond and half used their left hand. The probes remained on the computer screen until the participant responded or for 3000 ms. Then a number or a word appeared in the center of the screen (task 2 – see **Figure 1**) and participants had to determine whether the number/word was the correct answer to the equation/rhymed with the previous word or not and to press a matching key on the keyboard (1 for correct answer and 2 for wrong answer). After responding or after 4000 ms a black screen appeared and remained for 1500 ms (for illustration of the trials see **Figure 1**). Following this period of time, the next trial began.

The task contained six blocks, each comprised of one sample of each stimuli type (four equation levels, math related and neutral words). In order to avoid ongoing stress levels, each block was followed by a 1 min break, during which an aquarium film appeared on the computer screen. Overall, the task consisted of 96 trials and lasted about 45 min.

##### The Revised Mathematics Anxiety Rating Scale

Participants answered a Hebrew translated computerized version of the *MARS-R* (Plake and Parker, 1982), which is a shortened version of the MARS questionnaire (Richardson and Suinn, 1972) containing 24 items. We created the computerized version using an online Google form document, completed by participants after performing the experimental task. The computerized version allowed us, among other things, to make sure that participants did not miss any questions.

The questionnaire was designed to reflect the degree of anxiety experienced in a variety of math related tasks and situations, based on 5-point Likert scale (1- not nervous at all to 5- very nervous). In order to obtain the total score, we simply summed up the scores for all questions. Since the literature does not set a clear threshold that represents HMA levels and based on the methods of previous studies, a median score of 72 points and higher (obtained by giving a rating of 3 or higher for each question) was chosen as representing HMA levels.

## Results

### Probe Identification Task (Task 1) – Accuracy Rates

Accuracy rates for the probe identification task were very high following all types of primes (single digits: *M* = 0.97, *SD* = 0.05; double digits: *M* = 0.96, *SD* = 0.04; triple digits: *M* = 0.96, *SD* = 0.07; powers: *M* = 0.98, *SD* = 0.04; math word: *M* = 0.96, *SD* = 0.05; neutral word: *M* = 0.94, *SD* = 0.06).

### Solution Task (Task 2) – Accuracy Rates

Mean accuracy rates for deciding whether the number presented is the correct solution of the prime (i.e., task 2; see **Figure 1**) was very low in both the power (40%) and triple digit (30%) equations. Mean accuracy rates of all the other equations and words were higher than 80%. Since our aim was to have all participants mentally process the prime and to make sure that the primes contain meaningful math stimuli, we did not analyze the triple and power equation. This was done under the assumption that at some point participants ignored the triple digit and the power equation, as they were too difficult or complicated to solve mentally.

We then conducted two-way repeated measures ANOVA on the prime accuracy rates (task 2). This analysis included the Anxiety group (HMA or LMA) as the between-subject factor and Prime type (i.e., single digits, double digits, math word, neutral word) as the within-subject factor.

There was neither significant difference between the groups (*F* < 7) in accuracy rates nor interaction between Group and Prime type.

### Solution Task (Task 2) – Reaction Time

There was no significant difference between the groups (*F* < 10) in RTs nor interaction between Group and Prime type.

### Dot Probe Analysis – Reaction Time

A four-way repeated measures ANOVA was conducted on the probe’s mean RTs. This analysis included the Anxiety group (HMA or LMA) as the between-subject factor and Prime type (i.e., single digits, double digits, math word, neutral word), Congruency (prime and probe congruent, vs. prime and probe incongruent), and Operation (i.e., addition, subtraction, multiplication, and division) as within-subject factors.

Only trials, in which the probe was correctly identified, were analyzed.

Because Mauchly’s Test of Sphericity indicated that circularity could not be assumed, all of the following *F*-statistics are adjusted by the Greenhouse-Geisser correction.

The results revealed a main effect of Prime type [*F* = 31.8, *p* < 0.001, η^2^ = 0.55], such that RTs for probes presented after single digit equations were faster (*M* = 841.9, *SD* = 49) than after double digit equations (*M* = 958.4, *SD* = 51.8) and both were slower than probes presented after neutral words, which were processed faster (*M* = 689.4, *SD* = 35.5) than math words (*M* = 748, *SD* = 46.2). No other main effects were evident (e.g., main effect of Group *F* < 8 not significant).

**FIGURE 2:**
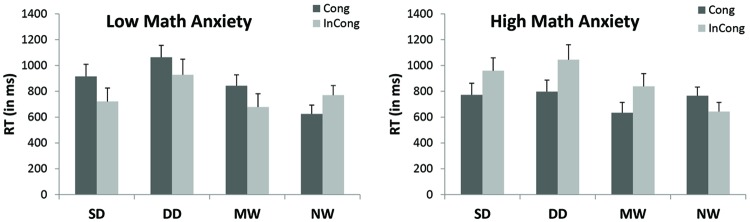
**Mean reaction times (RTs) of type of probe and congruency as a function of group (significant interaction between Group, Probe, and Congruency).** SD, single digit equations; DD, double digit equations; MW, math words; NW, neutral words. Cong, congruent (i.e., probe and prime are presented at the same location); InCong, incongruent (i.e., probe and prime are presented at opposite locations). Error bars denote the standard error of the mean.

The triple interaction between Group × Type × Congruency was significant [*F*_(3,69)_ = 3.77, *p* = 0.05, η^2^ = 0.16] (see **Figure 2**). We further conducted simple interactions of Group × Congruency separately for math related probes (i.e., single and double digits and math words).

#### Math Related Probes

The simple interaction between Group and Congruency was significant [*F*_(1,25)_ = 4.1, *p* = 0.05, η^2^ = 0.14]. The simple main effect of congruency was significant in the *HMA group* [*F*_(1,13)_ = 31.8, *p* > 0.001], indicating that congruent probes were processed significantly faster (*M* = 723 ms) than incongruent probes (*M* = 925 ms). This simple main effect of congruency was not significant in the *LMA group*.

#### Neutral Words

The simple interaction between Group and Congruency was not significant.

It is interesting to note that when analyzing the simple interaction of Group × Congruency in math words only, the interaction was significant [*F*_(1,25)_ = 4.2, *p* = 0.017, η^2^ = 0.14]. The simple main effect of congruency was marginally significant in the HMA group [*F*_(1,13)_ = 2.09, *p* = 0.17], indicating that congruent probes were processed faster (*M* = 627 ms) than incongruent probes (*M* = 814 ms). This simple main effect of congruency was not significant in the LMA group.

In an additional different analysis we looked at MA scores as a continuum. Specifically, in the current analysis we correlated MA scores (MARS) with the mean congruency effect (incongruent – congruent) for the math related trials. This correlation was found to be significant and positive [*r* = 0.4, *p* < 0.05], indicating that the higher the MA the larger the effect.

## Discussion

The appearance of biases in the cognitive processes of individuals with general anxiety has been highlighted as a distinction of the etiology, maintenance, and treatment of anxiety disorders (Beck and Clark, 1997). Specifically, there is accumulating evidence that anxiety is associated with a bias in early preattentive processes that are likely to be involved in initial orienting of attention toward threat stimuli. How do we go about linking this characteristic to the cognitive profile that defines MA? We identified here two possible accounts for clarifying the cognitive status of MA: (1) math is associated with negative valence, and (2) attentional bias is related to numerical information. Broadly speaking, these claims respectively indicate that for math anxious individuals, math related stimuli such as math words or math equations are cognitively or affectively linked with threatening and negative valence (Rubinsten and Tannock, 2010; Rubinsten et al., 2012). Accordingly, for math anxious individuals, the cognitive system selectively favors the processing of emotionally negative information (e.g., math related words). Though not directly measuring selective attention to numerical information, the previous findings of Rubinsten and Tannock (2010) and Rubinsten et al. (2012) pointed to selective attention to negative information and support current findings. Indeed, current findings show, as in the typical dot probe task, faster RTs in HMAs, when probes appear in the locus of the numerical prime (i.e., either single and double digit equations or math words). Such a congruency pattern (i.e., faster RTs for congruent than for incongruent trials) was not found in the case of neutral word primes; or at least, high math anxious individuals processed neutral words similar to low MA individuals. It is important to note that there was no significant main effect of RT between the two groups; HMAs was not generally slower. Moreover, there was no significant main effect of accuracy levels between the groups either for detecting the probe or for solving the math equations. Hence, HMAs did not show lower performance and did not need additional time in order to solve the tasks. That is, the longer time it took the HMA group to locate the congruent probe (compared to the incongruent) is due to the threatening affect associated with the math equation and not since these equations were too complicated to solve.

Several authors have tried to further differentiate between different components of attention (engagement, disengagement, and shifting – see Posner and Petersen, 1990) in the dot probe task (Koster et al., 2004, 2006; Salemink et al., 2007). However, and since the measurement of the separate components has been previously challenged, there is general agreement that the dot probe task is a useful measure of attentional bias as a single entity that includes all of these components. Hence, and because the focus of our study is attentional bias as a single entity in MA, we cannot reach a conclusion about the different components of attention. However, the long presentation time of the prime in the current study (1000 ms) may suggest that math anxious individuals show a general bias in cognitive processing, and hence, once their attention has settled on a threatening numerical stimulus, they have successive difficulty in disengaging it.

Specifically, Bradley et al. (1998) examined biases in initial shifting versus maintenance of attention, by manipulating the exposure duration of the threatening prime stimulus. Their results indicated that the attentional bias for threat was not significantly different between the two different exposure durations (500 and 1250 ms). Given that the duration of 1250 ms in Bradley’s study and 1000 ms in the current study potentially allow multiple shifts of attention, our results (based on the findings of Bradley et al., 1998) may suggest that attentional bias in anxiety operates in both initial orienting and in the maintenance of attention – math anxious individuals do not disengage attention from the negative stimulus. This view is compatible with Beck’s (1979) model, which suggests that anxiety related biases favoring threat stimuli operate on both attentional levels (i.e., initial orienting and maintenance of attention).

Attentional bias allows the cognitive system to prioritize specific stimuli for processing. Accordingly, responding to threats may in fact facilitate survival and learning. For example, mammals tend to learn mainly about those aspects of the environment to which they attend (for review see Shechner et al., 2012). Following this line of logic, it would be expected that math anxious individuals, who present attentional bias toward numerical contents, will show better learning curves and better math performance. This is of course not the case. We show no significant differences in accuracy rates between high and low math anxious individuals, and previous studies have shown low math performance in MA (e.g., Maloney et al., 2010; Rubinsten and Tannock, 2010). Accordingly, it may be suggested, although not directly studied here, that attentional bias is related to rumination, which directly impacts performance and significantly affects individuals’ ability to remain attentive to the task at hand (Donaldson et al., 2007). Indeed, Reese et al. (2010) suggested that attentional bias to negative information may be the factor that contributes to the pattern of distressing and repetitive negative thinking that characterizes anxious rumination and worry. Accordingly attentional bias and rumination in the case of MA, suggest constant obsessive thoughts over negative feelings related to math and the stress that mathematical problems cause, consequently turning attention away from the ways in which one can actually solve these problems (Ashcraft and Moore, 2009; Beilock and Ramirez, 2011).

It is important to note that, due to methodological limitations, the vital question of causality (between attentional bias and MA) cannot be answered here. This causality question is nevertheless crucial, not only from a scientific perspective but also from a clinical perspective. If cognitive and, specifically, attentional biases are causally involved in the development of MA, then therapeutic interventions should aim to reduce these cognitive biases to prevent or reduce the individual’s level of anxiety.

There are some additional limitations in the current study, such as small sample size or no information on general anxiety levels. However, the significant triple interaction between group, congruency, and type of equation may suggest that sample size was sufficient to answer the current research question. Importantly, though, it should be noted that participants in the current study were divided into high vs. low MA groups using a median split. Some argue that a median split to dichotomize the scores may not be the most valid method of assessing high or low levels of participants (Waller and Meehl, 1998). Hence, it might be claimed that our group selection criteria may not be the best to answer current research questions. This indeed might be the case and could be considered a limitation and yet it should be mentioned that several other studies in the field of MA used a similar criterion for different tests (e.g., 2 working memory groups, Beilock and DeCaro, 2007; Ramirez et al., 2015).

## Conclusion

The current findings show that math anxious individuals shift their attention toward numerical stimuli, which for them are associated with negative and threatening valence. That is, this study strongly implicates biased processing of threats in the maintenance of MA. Attention is highly relevant for several other cognitive processes, such as memory and other forms of learning. Hence, the study of attention biases appears particularly pertinent to MA research, as attention affects learning and, specifically, math learning.

## Conflict of Interest Statement

The authors declare that the research was conducted in the absence of any commercial or financial relationships that could be construed as a potential conflict of interest.
