# Supplementary material for: Attentional bias in math anxiety
Source: Front Psychol. 2015 Oct 16;6:1539. doi: 10.3389/fpsyg.2015.01539 (PMC4607867; doi:10.3389/fpsyg.2015.01539)
Supplement: Supplementary file 1 [file Data_Sheet_1.DOCX]

**Appendix 1. Dot probe - stimuli list (prime)**

*Numerical stimuli:*

| Single digits | Double digits | Triple digits | Powers |
| --- | --- | --- | --- |
| 8+2  8-2  8/2  8*2 | 52+13  52-13  52/13  52*13 | 492+123  492-123  492/123  492*123 | 8^2^+2^4^  8^2^-2^4^  8^2^/2^4^  8^2^*2^4^ |
| 8+4  8-4  8/4  8*4 | 46+23  46-23  46/23  46*23 | 536+268  536-268  536/268  536*268 | 9^2^+3^5^  9^2^-3^5^  9^2^/3^5^  9^2^*3^5^ |
| 6+2  6-2  6/2  6*2 | 64+32  64-32  64/32  64*32 | 945+315  945-315  945/315  945*315 | 2^5^+4^2^  2^5^-4^2^  2^5^/4^2^  2^5^*4^2^ |
| 6+3  6-3  6/3  6*3 | 96+48  96-48  96/48  96*48 | 872+436  872-436  872/436  872*436 | 4^3^+8^2^  4^3^-8^2^  4^3^/8^2^  4^3^*8^2^ |

*Word stimuli:*

**Math related words**

| Word  (translation) | Familiarity score (average) |
| --- | --- |
| Arithmetic | 9 |
| Mathematics | 9 |
| Multiplication | 8.9 |
| Addition | 9 |
| Subtraction | 9 |
| Division | 9 |
| Percentiles | 9 |
| Algebra | 8.9 |
| Geometry | 8.9 |
| Ruler | 9 |
| Quantity | 9 |
| Counting | 9 |
| Number | 9 |
| Enumeration | 8.7 |
| Roots | 9 |
| Unknown | 9 |

Note: familiarity score is based on average answer to the question "How familiar are you with the word" on 1 to 9 Likert scale (1- not familiar at all, 9- very familiar)

**Neutral words**

| Word (translation) | Familiarity score (average) | Frightening score  (average) |
| --- | --- | --- |
| Wall | 9 | 1.13 |
| Paper | 9 | 1.09 |
| Notebook | 9 | 1 |
| Plate | 9 | 1 |
| Closet | 9 | 1.05 |
| Table | 9 | 1.03 |
| Chewing gum | 9 | 1.07 |
| Shoes | 9 | 1 |
| Television | 9 | 1.17 |
| Drawer | 9 | 1 |
| Loudspeaker | 9 | 1.07 |
| Refrigerator | 9 | 1.05 |
| Suitcase | 9 | 1 |
| Key | 9 | 1.03 |
| Kite | 8.98 | 1 |
| Picture | 9 | 1.05 |

Note: familiarity score is based on average answer to the question "How familiar are you with the word" on 1 to 9 Likert scale (1- not familiar at all, 9- very familiar). Frightening score is based on average answer to the question "How frightening is the word"? on 1 to 9 Likert scale (1- not frightening at all, 9 – very frightening).
